# An Overview on Guidelines on COVID-19 Virus and Natural and Assisted Reproductive Techniques Pregnancies

**DOI:** 10.22074/ijfs.2020.46230

**Published:** 2020-10-12

**Authors:** Reihaneh Pirjani, Maryam Rabiei, Ameneh Abiri, Ashraf Moini

**Affiliations:** 1. Department of Obstetrics and Gynecology, Arash Women’s Hospital, Tehran University of Medical Sciences, Tehran, Iran; 2. Breast Disease Research Center (BDRC), Tehran University of Medical Sciences, Tehran, Iran; 3. Department of Endocrinology and Female Infertility, Reproductive Biomedicine Research Center, Royan Institute for Reproductive Biomedicine, ACECR, Tehran, Iran

**Keywords:** Corona Virus, COVID-19, Guideline, Pregnancy

## Abstract

In this article, we reviewed and compared some of COVID-19 and pregnancy guidelines; this can be useful for pregnant women including those with a history of infertility specially those undergone assisted reproduc- tive techniques (ART). The general advice given for prenatal care is to reduce face-to-face visits. All women who refer for prenatal visits should be evaluated for signs of the infection at the time of entry. The triage of suspected women should be done separately from other patients. Outpatient monitoring with a 14-day self- quarantine can be considered for asymptomatic infected women and for those with mild symptoms. Inpatient management criteria include moderate to severe symptoms and the target level of oxygen saturation is 92 to 95% in different guidelines. In the presence of fever, it is important to conduct a thorough examination of other causes of the fever. It is important to monitor fluid intake and output, maintain fluid and electrolyte balance and prevent fluid overload. Thromboembolic prophylaxis is recommended. Corticosteroid administra- tion is based on obstetrics indications, while in critical ill cases, it should be based on multi-disciplinary teams (MDT) decision. A positive COVID-19 result in the absence of other obstetrics causes, cannot be considered an indication for delivery in mild and asymptomatic cases. In critically ill pregnant women, an individualized decision should be made about delivery time by the MDT. General anesthetic should be avoided unless inevi- table for standard procedures such as intubation is an aerosol-generating procedure (AGP). There is agreement on the point that babies born to infected mothers, even if isolated from the mother at birth, should be consid- ered a close contact of the mother and tested for COVID-19 and separated from other neonates. Breastfeeding is encouraged and hand hygiene and face mask during feeding are highly recommended by all guidelines.

Over the past few months, COVID-19 caused by the coronavirus has become pandemic and has approximately affected all aspects of people's life around the world. The incubation period of COVID-19 infection is approximately estimated to be 5 to 6 days with a maximum of 14 days, and the virus can remain on some surfaces for up to 72 hours (1). In non-pregnant cases, it almost takes 2 week on average from appearance of symptoms to recovery, in mild cases and three to six weeks in severe cases (2) and mortality has been reported between 0.9 and 9% in different populations and countries (3). People around the world have made changes in their daily life and social interactions to reduce the risk of the virus transmission as much as possible. Along with other changes in daily life, all the medical guidelines in all disciplines including obstetrics, have been modified according to the new pandemic conditions.

To date, no effective treatment for the virus has been found, and even little is known about the effects of the virus and how to protect against it or prevent it. Given that there was no previous scientific evidence, researchers around the world began extensive research about the virus and even changed their research field to the virus (4-6) . Before this pandemic, the guidelines were prepared based on strong scientific documents, but in the case of COVID-19 pandemic, due to an inevitable need for new guidelines and lack of strong evidence, guidelines are being modified based on expert consensus or documents that do not have a strong scientific basis. It should be noted that since pregnant women especially those with a history of infertility are known as high-risk population during COVID-19 pandemic crisis, it is important to have a useful guide for pregnancy. So, in this article, we reviewed and compared some of the reliable guidelines in the field of pregnancy and coronavirus including American Society for Maternal-Fetal Medicine (ASMFM), The Royal College of Obstetricians and Gynaecologists (RCOG), Queensland Guideline, The Royal Australian and New Zealand College of Obstetricians and Gynaecologists (RANZCOG), The International Federation of Gynecology and Obstetrics (FIGO), and Government of Western Australia (1, 2, 7-12).

## All-purpose principles for prenatal care in all pregnancies in the period of COVID-19 pandemic

Physiological changes, immune suppression, and increased oxygen demands in pregnancy may impact the severity of symptoms and may cause some adverse outcomes (e.g. preterm delivery, restricted fetal growth and fetal and neonatal mortality) in pregnant women with lower respiratory tract infections (2), but based on the available data, it seems that pregnant women are not at higher risk compared to non-pregnant women, and the virus has not been found in the amniotic fluid, vaginal discharge, or breast milk until now (1, 8). The current findings do not indicated an increasing risk of first-trimester miscarriage and vertical transmission (1).

In the COVID-19 pandemic, priority should be given to preventing the spread of the virus, so pregnant women should be told to report any symptoms of a sore throat and fever before a face-to-face visit. If a pregnant woman has suspicious symptoms or has been in contact with an infected person, she should not enter the clinic so that their appointment can be changed. The general advice given for prenatal care in all guidelines is to reduce face-to-face visits and do only essential cares, for example, it is better to perform prenatal care and ultrasound examination on the same day and in one place with no or only one attendant to minimize patient and health care provider risks (1, 2, 7-10, 12).

An ultrasound examination in the first trimester is agreed by all guidelines. It is suggested to perform a single ultrasound between 11 to 14 weeks, to determine the gestational age and nuchal translucency (NT) in parallel with a combined screening test (2, 9, 10, 12).

ASMFM suggested that if somebody prefers to perform cell-free DNA testing, NT scan can be considered optional (10). All guidelines agree upon anomaly scan ultrasound at 18 to 23 weeks and an ultrasound examination at the third trimester to assess fetal growth based on risk factors. In all guidelines, phone or video call, electronic record system or telemedicine is a reasonable option that limits patient exposure. Queensland guideline has suggested that health care providers can also visit patients at homes (2). However, organizations that provide such care must be organized to plan patient visits and appropriate feedback. All guidelines agree upon adjusting the number of prenatal visits, but emphasize that any decision to adjust prenatal care should be made based on regional and individual circumstances and delayed visits should not affect the health and safety of pregnant women. COVID-19 outbreak potentially augments the threat of domestic violence (12). All guidelines emphasize that women should be investigated for mental health at all face-to-face or through telemedicine visits (1, 2, 7-10, 12). So seclusion, grief, economic drawbacks, lack of security and failure to access supportive apparatus, are all commonly acknowledged risk factors for being psychologically unhealthy (12). Women who refer for prenatal visits should be evaluated for signs of the infection at the time of entry, and tested if they have symptoms. They should be asked about fever and respiratory symptom and previous contact with infected people. In the presence of mild and moderate symptoms including new fever and new cough, they should be referred for COVID-19 test and in the presence of more severe symptoms including chest pain and shortness of breath, they should undergo hospitalized evaluation (11). Efforts must be made to provide an equilibrium regarding the risk of unnoticed maternal and fetal complications due to pre-existing co-morbidities against the possible dangers of COVID-19 (12).

## Approach for suspected or confirmed COVID-19 pregnancies

Women who are being admitted with any evidence of pneumonia, Acute Respiratory Distress Syndrome (ARDS), or temperature more than 37.8˚C and at least one of acute incessant coughing, shortness of breath, hoarseness, wheezing or sneezing nasal discharge or congestion, or sore throat, should be evaluated for COVID-19 (9). The triage of suspected women should be done separately from other patients, and it should be determined whether they need inpatient care or can be monitored on an outpatient basis (8). RCOG recommends sending a full blood count for women with an isolated fever; if lymphopenia is detected, testing for COVID-19 is highly recommended (12). Health care providers should attempt to restrict displacement of the patients from one place to another (11).

## Protocols for outpatient care

Outpatient monitoring with a 14-day self-quarantine can be considered for asymptomatic infected women and for those with mild symptoms (10). If outpatient monitoring is needed, the patient should go home by a private vehicle, stay at home, not attend public places such as work, school, or shopping centers, not see visitors and the house windows should be open and she should be separated from other family members. In addition to women with a positive COVID-19 test, self-isolation of 14 days is also recommended while waiting for the test result or being in touch with a confirmed case of COVID-19 (8). 

Outpatients women should be monitored closely and given specific instructions about when to contact their health care providers, they should perform daily self-assessments and obstetric care providers should ensure that the medical institution has a reliable feedback mechanism for early detection of a worsening condition (10). Urgent pre-arranged appointments for high-risk pregnancies will need an individual decision on urgency and potential risks or benefits. In cases where there is an urgent and unplanned need for appointment, it is better to call first and be guided by phone, and if it is deemed that prenatal care cannot be delayed until termination of the advised period of quarantine, it is better to provide facility for local care, ideally at the end of a working day (12). There is no guidance about the timing of occurrence for followup outpatient care; nonetheless, it is commonsensical to conduct a follow-up visit at least once within 2 weeks of diagnosis of COVID-19. These visits can either be carried out through telemedicine or specialized COVID-19 clinics where obtainable. Obstetric care providers should engage in outpatient care to screen for potential obstetric complications and maternal and fetal health (10). Routine prenatal cares such as fetal growth scans and Glucose Tolerance Test should be postponed until termination of the quarantine period. Fetal well-being tests such as Non Stress Test and Biophysical Profiles should be performed based on obstetrics indications and, if possible, should be reduced (10). Although the link between intrauterine growth disorder and coronavirus has not yet been established, but considering the data from SARS, RCOG recommends that pregnant women who become infected, have a fetal growth ultrasound two weeks after recovery (12). If a person is infected in the first trimester, although the data in this regard is not complete, she can have a detailed anomaly scan at 18-24 weeks (2, 8). As well, ASMFM recommends that those infected in the first trimester, should undergo a thorough anomaly scan, and those who become infected in the later stages of pregnancy, undergo an ultrasound scan in the third trimester (10).

## Protocols for inpatient care

Inpatient management criteria include moderate to severe symptoms, oxygen saturation below 95%, associated underlying diseases and fever higher than 39ºC despite taking acetaminophen. ASMFM has also mentioned that increases in work of breathing (respiratory rate greater than 30 bpm, use of accessory muscles, pursing of lips, and need for oxygen supplementation) are also important signs of worsening of the patient's condition; ASMFM has pointed to exertional oxygen saturation as a criterion for inpatient admission (10). Based on The International Federation of Gynecology and Obstetrics (FIGO) statement, hospitalization should be considered for pregnant women with fever and respiratory difficulties (7). The target level of oxygen saturation is considered 92-95% by Queensland guideline, above 94% by RCOG and above 95% by ASMFM. Patients with oxygen saturation during walking oxygen saturation test ≤95% on room air, should be considered for inpatient admission. If hospitalization is required, health care providers including midwifery nurses, obstetricians, anesthesiologists, and neonatologist, should be informed and visits should be limited. All guidelines recommend keeping the patients in negative pressure rooms if possible and isolate them in a room which has a place to change the clothes of the staff and has facilities such as a bathroom; also, personal protection is emphasized in contact of suspected or confirmed patients (1, 2, 7-12). All guidelines agree that suspected or confirmed COVID-19 cannot be the only indication for admission or transfer. However, in case of moderate disease, given the possibility of deterioration of maternal condition and preterm birth, early transfer should be considered and private transport is recommended where possible; during the transfer, the patient should have a face mask. Necessary imaging should be performed regardless of pregnancy and, of course, abdominal shielding should be used on the gravid uterus. Fetal monitoring should also be performed according to obstetrics indication and whenever fetal intervention, including delivery, fetal monitoring should be considered. Treatment and management of hospitalized patients should be done based on clinical symptoms.

When it is highly probable to have be a staggering number of patients infected with COVID-19, health care providers should bear in mind to consider other possible differential diagnoses and care requirements. It is of utmost importance to offer permanence and steadiness in maternity services, and stay alert and concentrated on the common roots of neonatal and maternal morbidity (1). In case of fever, it is important to conduct a thorough examination of other causes of fever and not consider all pyrexia related to COVID-19 (12).

It should be noted that in some cases, even in the presence of hypoxia, tachycardia may not develop; so, it is important to check vital signs, and oxygen saturation along with other vital signs whenever there is an indication (1, 2, 7-11). Frequency of vital sign assessment depends on the severity of illness so that for patients with mild symptoms, it can be performed every 4 to 8 hours and for whom with severe disease, every 2 to 4 hours. For patients with critical illness, continuous pulse oximetry and telemetry should be done and recording should be done every 1 to 2 hours; also, noninvasive and invasive cardiovascular monitoring can be considered if indicated. Patients should be treated based on their clinical symptoms. For the time being, no therapeutic or prophylactic medications for COVID-19 are approved by the U.S. Food and Drug Administration and there is no proven antiviral treatment (10). Several reports illustrated that even after a period of recovery, there might be a possibility of a quick worsening of the situation. Following a woman’s improvement, it is reasonable to evaluate heart at least about 24-48 hours later. On discharge, advice should be given to the woman to come back instantaneously whenever she feels any deterioration in her condition (12).

## Severe and critically ill patients

ASMFM defined Severe disease by an O_2_ saturation ≤93%, a respiratory rate higher than 30 bpm, a ratio of arterial partial oxygen pressure to fraction of inspired oxygen of <300, or >50% lung involvement on imaging and defined Critical Disease as shock, or respiratory insufficiency which needs mechanical ventilation or high-flow nasal cannula, and multi-organ failure (10). RCOG has emphasized that we should be careful about the symptoms that indicate decompression, such as the number of breaths above 30, need for oxygen above 40% or a decrease in urine output (12) . Deterioration of myalgias, unrelenting or more recurrent fevers, decreased oxygen saturation, deteriorated of dyspnea and increased work of breathing should be considered as early warning signs and in the presence of failure to sustain oxygen saturation .95% by supplemental oxygen, rapidly increasing supplemental oxygen need, hypotension (mean arterial pressure MAP <65 mmHg) despite adequate fluid replacement or any document of end-organ dysfunction (e.g. altered mental status, renal failure, hepatic failure, cardiac dysfunction, etc.), management in intermediate acuity setting or intensive care unit admission should be considered (10). Since thrombocytopenia may occur in critically ill COVID-19 patients, aspirin and thromboprophylaxis must be hold in cases who suffer from thrombocytopenia (12).

Intubation timing should be individually considered. Maternal status, preexisting co-morbidities, presence of multi-organ failure, vital oxygen supplementation, and need for transport to a facility with a higher level of care should be taken into account when inserting an ultimate airway. Use of noninvasive positive-pressure ventilation, e.g. Bilevel Positive Airway Pressure (BiPAP) or Continuous Positive Airway Pressure (CPAP) are some alternatives for intubation, but BiPAP and CPAP use are contentious due to concern for aerosolizing infectious particles, although some institutions have employed these modalities in order to evade intubation (10).

## Some medications in suspected or confirmed COVID-19 infected pregnancies

## Empiric antibiotic therapy

Attention should be paid to bacterial pneumonia co-infection. If community-acquired pneumonia co-infection is suspected, infectious counseling must be done (2, 8); the use of antibiotics is reasonable, culture data should be obtained before initiating antibiotics, and empirical antibiotic treatment may be done while waiting for the results and starting antibiotics should not be delayed for more than 45 minutes in case antibiotics are indicated. Ceftriaxone plus azithromycin or ceftriaxone alone that are commonly used, are not contraindicated in pregnancy (10) . Patients with elevated procalcitonin levels may have a bacterial co-infection. It should be noted that a high procalcitonin level does not exclude COVID-19 infection.

## Fluid administration

It is important to monitor fluid intake and output, maintain fluid and electrolyte balance and prevent fluid overload. The guidelines have stressed that invasive fluid therapy should not be performed (1, 2, 7-10) . In hypovolemic cases and NPO status, administration of maintenance intravenous fluids is recommended and in acute resuscitation, in order to avoid worsening of pulmonary edema, a conservative fluid therapy (500-1000 ml bolus of crystalloid fluids), should be considered (10). Since acute respiratory distress syndrome may accompany this infection, in the setting of moderate-to-severe symptoms of COVID-19, hourly monitoring of fluid input and output is reasonable. After 250-500 ml boluses and before proceeding with further volume resuscitation, fluid overload should be assessed and during labor, caution with neutral fluid balance is emphasized (12).

## Venous-thromboembolic prophylaxis

Besides pregnancy which is itself a risk factor for thromboembolism, infectious conditions, due to inflammatory mechanisms, stimulate coagulation; also, isolation of infected people leads to sedentary lifestyle and increased chance of coagulation. Thus, all guidelines emphasize on thromboembolic prophylaxis even in the absence of any risk factor. All patients hospitalized due to COVID-19 infection, have to receive anticoagulants except when they are waiting to give birth within the next 12 hours. Following birth, in the absence of postpartum hemorrhage, anticoagulant should be started as soon as possible, and in the setting of regional anesthesia, RCOG states that it should be started four hours after the last injection or after removing the catheter. Where complicated COVID-19 cases are receiving the care of multi-disciplinary teams (MDT), proper low-molecular-weight heparin (LMWH) dosing should be evaluated in an MDT including an obstetrician (12) and ASMFM states that when regional anesthesia is considered, anesthesiologists should be consulted regarding the timing of anticoagulation therapy (10) . According to RCOG, regardless of the delivery type, anticoagulants should be taken for 10 days after delivery. Clearly, risk assessment for venous thromboembolism should be done and if there is another indication to continue, anticoagulants should be continued based on the indication (12).

In critically ill pregnant women and mechanically-ventilated patients, given the increased risk of thromboembolic events, if there is no contraindication, prophylactic anticoagulation is highly recommended, but in terms of therapeutic anticoagulation, there is limited evidence. Moreover, the increased risk of preterm labor in inflammatory illness (spontaneous or iatrogenic) also can increase the risk of peripartum hemorrhage, where anticoagulant therapy may worsen the situation. Importantly, there are no clinical data to support if early, full-dose anticoagulation is advantageous in these patients; so, decision about therapeutic anticoagulation should be individualized (10) . For patients receiving prophylactic dosing, LMWH may be preferred due to the once-daily dosing to reduce exposures to health care providers.

Given the short half-life of unfractionated heparin and its reversibility with protamine sulfate, it is preferred in critically ill cases without confirmed thrombosis. As well, unfractionated heparin is preferred for prophylaxis in pregnancies at risk for preterm labor (10).

## Corticosteroid administration for fetal lung maturation

In case of confirmed or suspected infection, corticosteroid administration done based on obstetrics indications should not be changed, but in severe critical ill cases that require intubation and ICU, corticosteroids administration may cause deterioration in their condition and should be done based on MDT decision (2, 7, 8). ASMFM states that corticosteroids should be used with caution due to their potential worsening effect on pulmonary status and viral shedding, and the maximum age for administration should be 34 gestational weeks (10). According to RCOG guideline, corticosteroids for fetal lung maturation must be administered whenever indicated and there is no evidence suggesting it can lead to any damage in the presence of the virus. Obviously, immediate intervention for birth should be implemented for their administration (12).

## Magnesium Sulfate

According to some guidelines, no change should be made to its usual indications (2). ASMFM indicate that decision-making should be done based on each individual case, for example, in cases such as fetal neuroprotection, it should be given before 32 weeks. Magnesium sulfate is not contraindicated in women with mild or moderate symptoms (11). However, in patients who are critically ill, magnesium sulfate should be discontinued or the dose should be adjusted, especially if benzodiazepines were taken. Since it is not yet known whether magnesium sulfate can cause pulmonary edema or not, it is essential to conduct an accurate fluid intake and output monitoring (10).

## Aspirin

According to all guidelines, aspirin can be continued in cases of clinical indications, such as prevention of preeclampsia.

## Tocolytics

There is still no finding in favor of changing tocolytics indications, but it is recommended not to use betamimetics due to the possibility of exacerbation of maternal hypotension, tachycardia and pulmonary edema. The use of nifedipine may be advantageous in infected patients because of some resemblances between high altitude pulmonary edema and lung presentations in COVID-19. NSAIDs (e.g. indomethacin) administration to COVID-19 patients has raised concern, and it can be used be as an alternative of NSAIDs as tocolytic if indicated. Nonetheless, no adequate data is available to advocate that this utilization should be changed during this time.

## Mode and timing of delivery

## Delivery time

According to all guidelines, in mild and asymptomatic cases, a positive COVID-19 test without other obstetrics causes, is not a reason for delivery and mode of birth would not be affected by the virus, unless the patient’s respiratory condition needs immediate intervention for birth. Patients who are currently in quarantine, should undergo an individual evaluation about possibility of delaying a previously-scheduled caesarean birth, or induction of labor, but possibility of an urgent delivery must also be taken into account (12). For cases whose elective caesarean section or induction of labor cannot be postponed, optimal management of the respiratory situation should be established and the woman should be evaluated by the MDT (1). In asymptomatic or mildly symptomatic pregnant patients, positive for COVID-19 at 37 to 38 6/7 gestational weeks without other indications for delivery, expectant management is recommended until 14 days after a positive COVID-19 polymerase chain reaction (PCR) or until 7 days after being symptomatic and 3 days after disappearance of symptoms. This choice can result in reduced exposure of health care providers and the neonate to COVID-19 virus. In an asymptomatic or mildly symptomatic pregnant patient positive for COVID-19 who is at 39 gestational week or more, delivery can be a potential alternative to lessen the risk of worsening maternal status (10). Since the severe peak of symptoms may occur in the second week , it is important to propound delivery before that time in mild and moderate cases not requiring urgent care (11). Data regarding delivery timing and acute respiratory distress syndrome are insufficient and inconclusive.

In critically ill pregnant women, an individualized decision should be made about delivery time by the MDT, according to maternal and fetal condition and potential betterment of the woman following elective birth. Obviously, maternal well-being should always be preferred (12) In critically ill pregnant women, surgical and neonatal equipment for an urgent cesarean section and hemorrhage package including methergine, prostaglandin f2ل and misoprostol need to be accessible at the bedside and tranexamic acid, which must be refrigerated, should be available in the ICU. Decisions for delivery time need close correlation between the obstetricians and critical care teams and should be made based on gestational age, maternal status, contemporary pulmonary disease (e.g. cystic fibrosis, asthma, or sarcoidosis), critical illness and possibility of removing the ventilator mechanics. It should be noted that the need for mechanical ventilation alone is not an indication for pregnancy termination. The uterus pressure during third trimester of pregnancy, can effect lung function including expiratory and inspiratory reserve volume, and functional residual capacity and can consequently cause severe hypoxemia specially in critically ill pregnant patients; so, theoretically, improvement of lung function can be achieved by early delivery and it is logical to think about delivery in the setting of worsening critical illness. After 34 gestational weeks, delivery should be considered for critically ill women, because delivery can lead to optimize respiratory condition (11) . Nevertheless, it is unknown whether delivery results in a substantial improvement of all case. So, if pregnancy termination is considered according to severe hypoxemia, particularly in pregnancies before 30-32 gestational weeks, other options including prone positioning, and even advanced ventilator methods such as extracorporeal membrane oxygenation (ECMO), should also be considered (10).

## Delivery mode

Decisions about delivery mode should also be made based on obstetric indications (1, 2, 7-10). However, in the absence of indications, normal vaginal delivery should be encouraged (1). If elective caesarean has been considered, it is important to individually evaluate urgency and the possibility of continued pregnancy by use of frequently fetal surveillance (2, 8). It is necessary to keep in mind that in the virus pandemic, most of pregnant women including those with history of infertility specially who underwent assisted reproductive techniques may be stressful about their childbirth. RCOG recommends that delivery mode should be explained for the women and their preferences should be taken into account (12). Based on the available data, there is no evidence about priority of delivery mode. Therefore, delivery mode should not be affected by the presence of the virus, unless the patient’s respiratory situation requires immediate delivery.

Since intubation is AGP, general anesthetic must be kept away unless it is obligatory for standard indications (2, 8).

## Intrapartum care

When a COVID-19 infected woman is hospitalized for childbirth, all the team members including neonatologist, obstetrician, midwife-in charge, neonatal nurse in charge, anesthetist and infection control team, should be notified. The time of birth should be noticed to neonatal team to allow them performing personal protective equipment (PPE)(1, 2, 7-10, 12). PPE contains gown, gloves, mask, pinafore, a fluid-resistant surgical gown, and a visor (1) . A negative pressure room for childbirth is preferred. Only one birth support person should be present during birth while wearing surgical mask (8). Low-risk COVID-19 infected women whose home is close to the hospital and have a private vehicle available, may prefer to stay at home in the latent phase of labor (1, 12). The woman should be given surgical mask on presentation; for the first stage of labor, she should be managed under contact and droplet precautions but for the second and third stages, using contact and airborne precautions is recommended if accessible (8) . The number of staff entering the room should be reduced to minimum (12). If it is possible to apply telemetry to provide a safe situation, the 2nd staff is allocated outside the room for double checking medications with the staff in the birth room (2, 8) . Patients should be allowed to have a single, asymptomatic birth partner during their labor. It is necessary that birth partners remain by the woman’s bedside, not walking around the ward and wash their hands repeatedly (11, 12). In addition to routine maternal management and standard practice, monitoring respiratory rate and oxygen saturations should be done. RCOG recommends hourly measurement of oxygen saturation, keeping O_2_ saturation >94% by O_2_ therapy (12) . Since there is no evidence that intrapartum oxygen therapy is beneficial for the fetus, and considering the point that face mask can be impregnated with the patients' respiratory secretions leading to treatment staff contact with the secretions, in the current situation, it is recommended not to use oxygen therapy even though nasal cannula and face mask are not recognized as an APG. Due to the high rate of asymptomatic COVID-19 carriers, this advice should be applied to all women during delivery. Early amniotomy and oxytocin augmentation to prevent dysfunctional labor can be considered. In the second stage of labor, women should be encouraged to push to avoid prolonged labor. Some actions such as massage of perineal body and using warm packs can lead to reduction of the third and four degree lacerations (11). When hypoxia or worn-out occur in a symptomatic woman during labor, individualized decision should be made with regard to using elective instrumental delivery in order to shorten the second stage of labor.

If symptoms worsen, an individual evaluation should be performed to make a decision about carrying out emergent cesarean or continuing the labor especially whenever it is possible to help the effort for resuscitation (12). Due to restricted resources for blood product, it is recommended to manage the third stage of labor by using prophylactic drugs including misoprostol and tranexamic acid to prevent postpartum hemorrhage (11).

In case of fetal compromise or distress, continuous fetal heart monitoring should be done (1, 2, 7-10). For asymptomatic COVID-19 infected women, continuous monitoring of fetal heart is not indicated only because of viral infection, unless in the presence of other medical indications (12).

Australian Guidelines recommend to beware of Fetal Scalp Electrode Monitoring and Fetal Blood Sampling until more data become available. However, if Fetal Scalp Electrode or Fetal Blood Sampling is planned, meditate on the potential hazard of fetal transmission against acknowledged merits of improved assessment of fetal condition (2, 8). However according to RCOG guideline, Fetal Scalp Electrode Monitoring and Fetal Blood Sampling can be performed and they are not contraindicated (12).

In cases where the woman develops a fever, in addition to considering COVID-19, other causes of fever and sepsis should be investigated according to guidelines on sepsis in pregnancy. Inexplicable fever caused by other reasons during labor or immediate postpartum should be assessed in a typical manner. However, it is recommended that a pregnant women should also be tested and/or monitored for COVID-19 according to institutional policy and guidelines (10). No document indicated that water immersion is contraindicated (2, 8). During the first stage of labor, women can be allowed to be in water (11), but according to some guidelines, since SARS-COV-2 has been detected in stool and accessible PPE is not generally waterproof and there is a potential risk of damage to PPE completeness during urgent procedures, using birthing pools in labor ward should be avoided in COVID-19 infected women (2, 8, 12). According to RCOG, labor and birth in water are not allowed for symptomatic COVID-19 infected women, however, data is inadequate for COVID-19-positive women who are asymptomatic (12).

## Pain relief

In terms of regional analgesia, no document showing regional analgesia is contraindicated in COVID-19 infected patients, is available (1, 2, 7-10) ; even epidural analgesia is recommended during labor for COVID-19 infected women given the possibility of urgent cesarean section and to avoid of general anesthesia as an AGP procedure (11, 12) . In terms of nitrous oxide, current information is not sufficient and there is a conflict about filtering, washing and AGP potential in the setting of COVID-19 (2). There is no data that Entonox using is an AGP. According to RCOG, when Entonox is considered, it is important to use a single-patient microbiological filter (12) but according to Queensland and Western Australia guidelines, for healthcare staff protection, administration for suspected or confirmed COVID-19 women should be avoided; however, it should be kept in mind that since asymptomatic women may ask to use analgesia during labor, if nitrous oxide is suggested, face mask instead of mouth piece should be considered(2, 8), and a microbiological filter of <0.05μm pore size should be used (8).

## Newborn care

There is no data indicating that delayed cord clamping can lead to increased risk of neonatal infection. When there is no other contraindication, delayed cord clamping is still reasonable because there is no contrary finding until now (1, 2, 8, 12). Cleaning and drying the baby is recommended as usual while the cord is still intact (12). According to RANZCOG, mothers and babies can practice skin-to-skin contact. Whether or not the mother has COVID-19 infection, she and her baby should be together all the time, even without delay after the birth, in order to establish breastfeeding (1). On the other hand, Centers for Disease Control (CDC) suggests that the mother and the baby should be separated from each due to the possibility of neonatal morbidity from maternal transmission (11).

The possibility of vertical transmission has not been completely excluded and even some evidence proposed the possibility of vertical transmission (12); also, intrapartum transmission due to exposure to maternal stool is probable and even mother’s respiratory secretions can be potential source of infection for newborns. There is agreement that babies born to infected mothers, because of the close contact with the mother even if isolated at birth, should be tested for COVID-19 and separated from other neonates. Although maternal infection alone is not an indication for neonatal admission, 14 days of quarantine in a shared location with mother is recommended for them. Surely, decision about mother and baby co-location should be made individually (2, 8, 10). However, FIGO has mentioned that according to some recommendations, neonates should be separated from their infected mother and kept in separate rooms or ≥6 feet away from the mother or physical barriers should be used (7).

## Breastfeeding

Since COVID-19 virus has not been found in breast milk until now, breastfeeding is not contraindicated (2, 7, 10). Really, as a potentially important source of antibody for the baby, breastfeeding is encouraged (7, 10). Shedding infective airborne droplet from the mother is probably the main risk for babies, so hand hygiene and wearing a facemask during feeding, are highly recommended by all guidelines. According to some guidelines, given the lack of enough evidence in this context, mother or baby skin washing is not necessary before close contact or breastfeeding (2, 8) while other guidelines recommend that before expressing breast milk, women should wash not only their hands but also breast (11). It is recommended to provide to current sterilization standards for breastfeeding equipment and formula preparation (2, 8).

## Discharge

It is recommended to consider normal discharge criteria. Also, it is suggested to notify the patients about continuing quarantine until completion of 14 days. For discharge home, a negative test is not mandatory. If discharged before completion of 14 days, constant clinical monitoring is advisable until 14 days. It is important to use local capacity such as telemedicine services and home visiting for clinical evaluation after discharge. Necessary advice should be given to the patient about indication for readmission. Most frequent reasons for readmission are respiratory symptoms one to three weeks after discharge (2, 8). Rapid deterioration has been reported even after a period of recovery, so, after improvement of a patient’s condition, at least 1-2 day followup should be performed; also on discharge, the woman should be enforced to return immediately if she feels bad.
